# Supplementary material for: The interactome of intact mitochondria by cross-linking mass spectrometry provides evidence for coexisting respiratory supercomplexes
Source: Mol Cell Proteomics. 2017 Dec 8;17(2):216–32. doi: 10.1074/mcp.RA117.000470 (PMC5795388; doi:10.1074/mcp.RA117.000470)
Supplement: Supplemental Data [file supp_RA117.000470_133922_0_supp_23978_fzffwf.zip › spectra_annotation/mito_spectra_annotation/161-1-1-1-24-1.pdf]

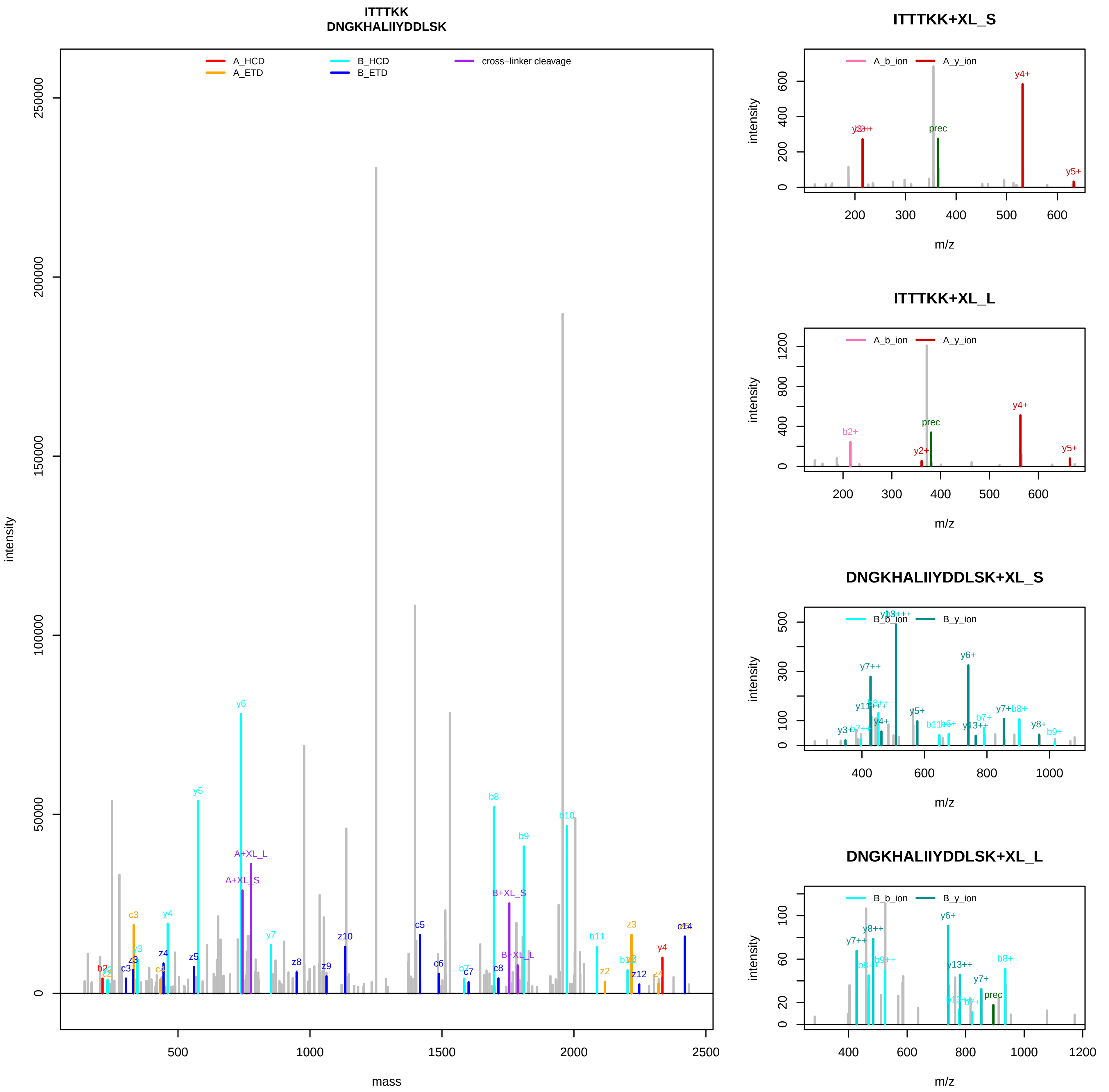

ITTTKK
DNGKHALIIYDDLSK
A_HCD
A_ETD
B_HCD
B_ETD
cross−linker cleavage
intensity
mass
0
50000
100000
150000
200000
250000
500
1000
1500
2000
2500
b2
c2
c3
z3
y3
z4
c4
y4
y5
z5
y6
A+XL_L
A+XL_S
y7
z8
z9
z10
c5
c6
b7
c7
b8
c8
B+XL_S
B+XL_L
b9
b10
b11
z2
z3
z12
y4
c14
ITTTKK+XL_S
A_b_ion
A_y_ion
y4+
y5+
prec
m/z
ITTTKK+XL_L
b2+
y2+
DNGKHALIIYDDLSK+XL_S
B_b_ion
B_y_ion
y13+++
y7++
y6+
y5+
y4+
y3+
y7+
b8+
y8+
b9+
y13++
DNGKHALIIYDDLSK+XL_L
y8++
y7++
b9++
b8++
y6+
y13++
y7+
b8+
prec
